# Virtual Dual inhibition of COX-2 / 5-LOX enzymes based on binding properties of alpha-amyrins, the anti-inflammatory compound as a promising anti-cancer drug 

**DOI:** 10.17179/excli2016-164

**Published:** 2016-03-23

**Authors:** Mohammad Mehdi Ranjbar, Vahideh Assadolahi, Mohsen Yazdani, Donya Nikaein, Behnam Rashidieh

**Affiliations:** 1 Razi Vaccine and Sera Institute, Karaj, Iran; 2 Cellular and Molecular Research Center, Kurdistan University of Medical Sciences, Sanandaj, Iran; 3 Laboratory of Bioinformatics and Drug Design, Pars Silico Bioinformatics Institute, Tehran, Iran; 4 Academic Center for Education, Culture and Research, Tehran, Iran

**Keywords:** drug discovery, alpha-amyrins, anti cancer drug, Cyclooxygenase-2 (COX2), 5-Lipooxygenase (5-LOX)

## Abstract

**H**ydro-alcoholic fruit extract of *Cordia myxa* was considerably effective on curing acute inflammation in mouse model. Previous studies suggested significant anti-inflammatory activities as well as potential anticancer agent of α-amyrins in seeds. Inhibition of Cyclooxygenase-2 (COX-2) and 5-Lipooxygenase (5-LOX) is significant in cancer prevention and therapeutics although this inhibition with chemo-drugs has its own side-effects. It is shown that these enzymes pathways are related to several cancers including colon, breast and lung cancer. This study was conducted based on Cordia species' α-amyrins as a safer natural anti-cancer compound for inhibition of COX-2 and 5-LOX enzymes by molecular docking. The X-ray crystal structure of COX2 / 5-LOX enzymes and α-amyrins was retrieved and energetically minimized respectively. The binding site and surface of enzymes were detected. Docking studies were performed by AutoDock 4.2 using Lamarckian genetic algorithm (LGA). Finally drug likeness, molecular pharmacokinetic properties and toxicity of α-amyrins was calculated. Molecular Docking revealed hydrogen and hydrophobic interactions between α-amyrins with both active sites of COX-2 and 5-LOX enzymes. Interestingly, it covalently bonded to Fe cofactor of 5-LOX enzyme and chelated this molecule. Base on binding energies (∆G) α-amyrin has more inhibitory effects on 5-LOX (-10.45 Kcal/mol) than COX-2 (-8.02 Kcal/mol). Analysis of molecular pharmacokinetic parameters suggested that α-amyrins complied with most sets of Lipinski's rules, and so it could be a suitable ligand for docking studies. Eventually, bioactivity score showed α-amyrins possess considerable biological activities as nuclear receptor, enzyme inhibitor, GPCR and protease inhibitor ligand. These results clearly demonstrate that α-amyrins could act as potential highly selective COX-/5-LOX inhibitor. Also, it is a safe compound in comparison with classical non-steroidal anti-inflammatory drugs (NSAIDs) that are known as cancer preventive agents, since it is free of side effects on human body and it can be a promising drug for cancer therapeutics.

## Introduction

Now it is widely accepted that COX-2 plays a pivotal role not only in inflammation but also in tumor-genesis. It is indicated that COX-2-selective inhibitors can be a novel class of therapeutic agents for colorectal polyposis and cancer (Sano et al., 1995[21]; Oshima et al., 1996[17]). COX-2 contributes to tumor formation and its growth. Both tumor and stromally derived COX-2 could influence tumor angiogenesis and immune function. Furthermore, COX-2 over-expressed in half of benign polyps and 80 - 85 % of adenocarcinomas and inhibitors of cyclooxygenase are preventive for colon cancer (Williams et al., 1999[24]). Thus, several studies indicate that nonsteroidal anti-inflammatory drugs reduce the risk of colon cancer. 

Moreover, the findings suggest that an increase in COX-2 expression associated with the development of breast cancer (Harris et al., 2000[9]) as well as lung cancer and possibly with acquisition of an invasive and metastatic phenotype (Hida et al., 1998[10]). The chemo preventive activity of NSAIDs against breast cancer provides the first evidence that cyclooxygenase-2 blocking agent, celecoxib-α specific COX-2 inhibitor suppressed the incidence, multiplicity, and size of malignant breast tumors, possesses strong chemopreventive activity against mammary carcinogenesis (Harris et al., 2000[9]).

On other hand, it is confirmed that LOX pathway receptor expression was found in the majority of cancers evaluated specifically in ovarian cancer. Dual 5-LOX/COX inhibitors are potential new drugs since they act by blocking the formation of both prostaglandins and leucotrienes but do not affect lipoxin formation. Such combined inhibition avoids some of the disadvantages of selective COX-2 inhibitors and spares the gatrointestinal mucosa (Martel-Pelletier et al., 2003[14]). Recently, it was indicated that dual inhibition of 5-LOX and COX-2 suppresses colon cancer effectively (Ye et al., 2005[25]). The most of chemical inhibitors which inhibit COX-2/5-LOX have various side effects including gastrointestinal ulcerogenic activity and bronchospasm. 

Inflammation, however, linked to cancer as tumors often arose at sites of chronic inflammation and that inflammatory cells were present in biopsied samples from tumors. Inflammatory diseases increase the risk of developing many types of cancer and non-steroidal anti-inflammatory drugs reduce the risk of developing certain cancers (such as colon and breast cancer) and reduce the mortality caused by these cancers (Mantovani et al., 2008[13]). In the light of these facts inhibition of an inflammation as a cancer inducer might have a role as important as cancer treatment.

Arachidonic acid (AA) pathway plays key roles in inflammatory response in which AA is oxygenated by the enzymes cyclooxygenase (COX) and lipooxygenase (LOX) and is then transformed into a variety of products which mediate or modulate inflammatory reactions (Martel-Pelletier et al., 2003[14]). There are two distinct established isozymes (COX-1 and COX-2) for COX. COX-2 is primarily responsible for inflammation (Julémont et al., 2004[11]; FitzGerald and Patrono, 2001[5]). Activity of 5-LOX enzyme in body produces Leucotriene compounds that contribute in inflammatory process as well (Julémont et al., 2004[11]; Pelletier et al., 2001[19]; Martel-Pelletier et al., 2003[14]). Inhibition of COX-2 and 5-LOX has been considered an effective mechanism for inflammatory preventions (Martel-Pelletier et al., 2003[14]). Inhibitors of both COX-2/5-LOX enzyme are known as effective promising regimes in controlling molecular processes of inflammation and showed minimal adverse effects compared to non-steroidal anti-inflammatory drugs (NSAIDs) (Julémont et al., 2004[11]).

In comparison to new drugs, herbal medicines and their relative compounds are frequently used to treat different inflammatory conditions, such as lung and skin inflammations as well as chronic diseases (Dixit and Mittal, 2013[3]; Garg et al., 2012[6]). Recently, we done an in-vitro study on anti-inflammatory properties of *Cordia myxa* fruit and results showed that the hydro-alcoholic extract was considerably effective in treatment of acute inflammation in rat (Ranjbar et al., 2013[20]). Cordia species plants are increasingly being referred to as a rich source of anti-inflamatory compounds (Ficarra et al., 1995[4]; Dixit and Mittal, 2013[3]). Moreover, several studies on Cordia species revealed analgesic, anti-inflammatory and other properties of different part of this plant (Ficarra et al., 1995[4]; Al-Awadi et al., 2001[1]; Shahapurkar and Jayanthi, 2011[22]). It contains phytochemicals which have immunomodulatory activity. Previous in vitro studies suggested that significant anti-inflammatory activity of seed is due to its active ingredients such as a-amyrins and taxifolin-3,5-dirhamnoside (71.4 %, 67.8 % respectively) (Nariya et al., 2011[16]). 

Isolation, characterization and screening of *in silico/in vitro* pharmacokinetics of active compounds from medicinal plants is common today and drug discovery techniques such as molecular modeling and docking are widely used to elucidate their effects on body (Geysen et al., 2003[7]). Ligand binding interactions with the receptor (especially protein targets) are central pathway to numerous biological process and designing therapeutic agents (Morris et al., 2009[15]). In this paper, we report the screening of α-amyrins as natural potent anti-cancer as well as anti-inflammatory compounds derived from Cordia species seed for dual inhibition of COX2 and 5-LOX enzymes by molecular docking and evaluate its drug likeness and toxicity, to study the binding pattern and characteristics of the molecule for application as a novel drug in treatment of inflammatory diseases.

## Methods

### Molecular structures and energy minimization

The X-ray crystal structure of Cyclooxygenase-2 (COX2) (PDB ID 4PH9) and 5-lipoxygenase enzyme (PDB ID 3vv9) were retrieved from protein data bank (www.pdb.org). Then, water molecules were removed and the enzymes cleaned from any unwanted interactions. Two selected structure were energetically minimized by SPDV viewer software tool with GROMOS96 implementation. The 3D chemical structure of α-amyrins (CID: 73170) was retrieved through the PubChem-compound database at NCBI webserver, USA (www.pubchem.ncbi.nlm.nih.gov). Also, Ligand (α-amyrins) was minimized by ChemAxon software.

### Preparation of receptors for docking

First, for correct ionization and tautomeric states of amino acid residues, all nonpolar hydrogens were merged (removed) and partial atomic charges were assigned using the Gasteiger-Marsili method. Then charges were added to enzyme structures and Kollman United Atom charges and atomic salvation parameters were assigned. In continue incomplete side chains replaced using Dunbrack rotamer library by Chimera 1.8 software. The binding site and surface of enzymes were detected based on the previous reported data beside the catalytic domain of the enzyme just near the metal ion Fe in both structures (Gilbert et al., 2011[8]; Wang et al., 2010[23]).

### Molecular docking

Molecular docking was carried out in order to evaluate a possible binding mode between alpha-amyrin and COX2 and 5-LOX active sites. Docking in virtual-screening gives suitable indication of the possible biological activities of compounds and reduces cost and time of drug discovery. It also estimates the strength of the binding, the energy of the complex and calculates the binding affinity using scoring functions. 

The docking studies were performed using AutoDock 4.2. The Lamarckian genetic algorithm (LGA), considered one of the best docking methods available in AutoDock, was selected to perform the molecular docking studies (Morris et al., 2009[15]). AutoDock has reported to have high accuracy in predicting binding free energies between ligand and receptor by considering that the receptor is rigid while ligand is free to move, with a comparatively low standard error. Therefore, COX2 and 5-LOX conformational flexibility was neglected by rigid receptor docking. All rotatable bonds were assigned for the ligands (Morris et al., 2009[15], Cosconati et al., 2010[2]). To assign the perfect grid of each ligand, grid box values were obtained by trial and error and from previous research using AutoGrid. Grid maps with 126 -126- 126 points (the x, y and z directions) were made and the grid-point spacing was considered 0.375 Å. Each map was centered such that it covered the entire protein especially all possible binding sites. The number of docking independent runs was set to 50 with the step sizes of 0.2 Å and 5°.

Final docked conformations (20 conformers for each docking) were clustered based on a tolerance of 1 Å all-atom RMSD from the lowest-energy structure. The docked conformations of α-amyrins were ranked into clusters based on the binding energy and the most favorable binding conformation with the lowest free energies was selected as the binding pose and visually analyzed. Hydrogen bonding and hydrophobic interactions between α-amyrins and COX2 and 5-LOX were analyzed using Auto Dock Tools and Chimera 1.8 software.

### Drug likeness and toxicity evaluation of the Ligand

Molinspiration server (www.molinspiration.com) was used to calculate the α-amyrins drug likeness and molecular pharmacokinetic properties. Drug likeliness indexes assist to the ideal procedure of rejection nonviable ligand compounds before they are even synthesized. Synthetic and natural compounds using as drug against diseases need to under the rules and conditions. Drug likeness may be defined as a complex balance of various molecular properties and structure features which determine whether particular molecule is similar to the known drugs.

This tool was used to perform prediction of bioactivity score for the most important drug targets as well as QSAR (Structure-activity relationship) studies in order to identify potential activators of biological targets. It offers free online services for calculation of important molecular properties (logP, polar surface area, number of hydrogen bond donors and acceptors).

Lipinski's Rule of Five was used to evaluate drug likeness, or determine if a chemical compound with a certain pharmacological or biological activity has properties that would make it potentially applicable as an oral drug. The rule describes molecular properties important for a drug's pharmacokinetics in the human body, including their absorption, distribution, metabolism, and excretion ("ADME”). Lipinski's rule considers a compound as a drug if it satisfies the following criteria: not more than 5 hydrogen bond donors (number of hydrogen bond donors ≤ 5), not more than 10 hydrogen bond acceptors (number of hydrogen bond acceptors ≤ 10), A molecular mass not greater than 500 Daltons (molecular weight ≤ 500), An octanol-water partition coefficient log P not greater than 5 (logP ≤ 5), Molecules violating more than one of these rules may have problems with bioavailability (Lipinski et al., 2001[12]). The compound that had more than one violation from these rules should be eliminated from the study (docking) because of bioavability problems.

The Lipinski's rule of five parameters and total polar surface area (TPSA) have shown to correlate with drug absorption.

Molinspiration tools analysis Lipinski's rule of five, these molecular properties are logP, polar surface area (TPSA), molecular weight, number of atoms, number of O or N, number of OH or NH, number of rotatable bonds, volume, drug likeness includes G protein coupled receptors (GPCR) ligand, ion channel modulator, kinase inhibitor, nuclear receptor and enzyme inhibitor ligand, and eventually number of violations to Lipinski's rule for ligand. 

Furthermore, bio-safety of the α-amyrins molecule inside the human body was also explored. Toxicity analysis was done by taking advantages of Toxtree v.2.6.0 software.

## Results

**Figure 1:**
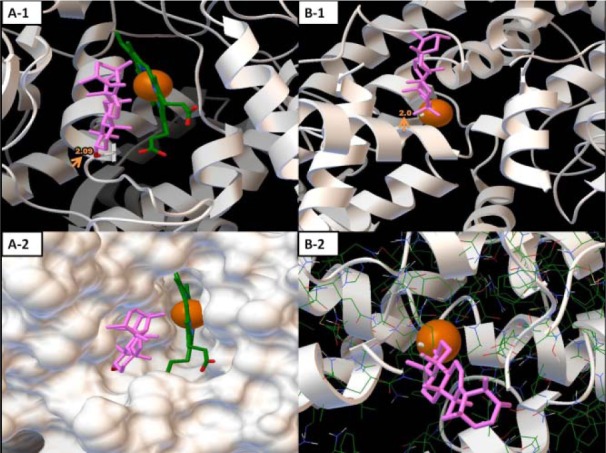
Figure 1: The binding modes of α-amyrins in the allosteric binding site pocket of COX-2 (A1 and A2) and 5-LOX (B1 and B2) enzymes. Arrows in A1 and B1 figures show hydrogen bonds between ligand and active sites.

Molecular Docking revealed interaction between α-amyrins with both active site of COX-2 and 5-LOX enzyme. α-amyrins interactions was stabilized by single hydrogen bond within the active site of amino acid GLN290 of COX-2 (Figure 1(Fig. 1): A1 and A2) and ASN554 of LOX-5 (Figure 1(Fig. 1): B1 and B2), and besides hydrophobic interactions.

Inspections of the active site pockets in both enzymes revealed it is located near Fe. Interestingly, α-amyrins directly bind to Fe cofactor of 5-LOX enzyme (by covalent bond) and it may chelate this molecule. In case of COX-2, α-amyrins is closely located near Hem.

Docking study of α-amyrins on binding sites of 5-LOX and COX-2 shows that ligands are good 5-LOX/COX-2 inhibitor and α-amyrins could be considered as promising inflammatory inhibitors of natural origin. Also, inspection of binding energies and interaction between cofactors (Fe or Hem) revealed α-amyrins has more inhibitory effects (binding affinity) on 5-LOX enzyme than COX-2 (Table 1(Tab. 1)).

### Results of drug likeness and toxicity estimations

Molecular physicochemical and the drug likeness are the two most significant properties to be considered for a compound to become a successful drug candidate.

Analysis molecular pharmacokinetic parameters are arranged in Table 2(Tab. 2). These results suggested that α-amyrins complied with the most of the Lipinski's rules (as it described in method) and has low polar surface area (PSA). However, LogP parameter is greater than 5 and violate from the rules. Term of LogP value shows the hydrophilicity character (lipophilicity) of compound and greatly affects absorption or permeation.

So, α-amyrins might desirably have passed through the Lipinski's rule of five criteria if some structural modifications occur **to improve** its membrane penetrability, although some successful commercial drugs do not follow some Lipinski's rules. Since α-amyrins rule number 5 violations was only seen in one parameter, thus this ligand could be checked for Docking Studies.

Bioactivity score of α-amyrins against GPCR ligand, ion channel modulator, kinase inhibitor, nuclear receptor ligand, protease inhibitor and enzyme inhibitory activity were evaluated and summarized in Table 3(Tab. 3). The molecule having bioactivity score more than 0.00 is likely to possess considerable biological activities, values -0.50 to 0.00 are expected to be moderately active and if score is less than -0.50, it is presumed to be inactive . With regard to bioactivity score, α-amyrins possess considerable biological activities as nuclear receptor, Enzyme inhibitor, GPCR and protease inhibitor ligand, respectively.

Toxicity of novel drug candidate is one of the major reasons for failure of drug discovery and screening efforts. Finally, toxicity analysis α-amyrins were subjected to toxicity assessment study by using Toxtree v.2.6.0. α-amyrins found to be free from high risks of undesired effects.

## Discussion and Conclusion

Although NSAIDs (such as aspirin, ibuprofen, naproxen) showed significant efficacy as anti-inflammatory and analgesic agents for treatment of inflammatory diseases and have profound effects on cancer as a preventive agent, long-term administration of NSAIDs lead to side-effects including renal failure and gastrointestinal (GI) symptoms including: mucosal lesions, bleeding, peptic ulcer, and intestinal inflammation which causes perforation and strictures in small and large intestines. In contrast, the 5-LOX/ COX-2 inhibitors profile showed they are free of gastrointestinal toxicity (Martel-Pelletier et al., 2013[14]). *Cordia spp.* and several plant extracts and their isolated compounds showed successful anti-inflammatory activity (Dixit and Mittal, 2013[3]; Ranjbar et al., 2013[20]) and they can be useful as cancer prevention or drug either (Parisotto et al., 2012[18]). 

In the present study, α-amyrins assessed for its anti cancer and anti-inflammatory activities using molecular docking with COX-2/5-LOX enzymes and also drug likeness and toxicity analysis. This compound exhibited significant anti cancer and anti-inflammatory activity, by inhibition of both COX-2 and 5-LOX enzyme. Its binding affinity with 5-LOX (∆G=-10.45 Kcal/mol) was more than COX-2 (∆G=-8.02 Kcal/mol), and it also covalently bonded to Fe. Also, hydrophobic interactions and hydrogen bonds between GLN290 and ASN554 with ligand were found in COX-2 and LOX-5, respectively.

The results of docking investigation suggest a good binding interaction which explains the significant biological activity of α-amyrins. Cordia extract by its active compounds could be a safe herbal alternative to COX-2/5-LOX inhibitor drugs for both cancer and inflammation, and offers a number of advantages such as lower side effects on human body and lower cost. 

Moreover, blocking the COX-2 enzyme completely is harmful for cardiovascular health and blocking a part of COX-2 activity is favorable. By respect to binding energy, hydrogen bonds and drug likeness of α-amyrins, it may partially block this enzymes and relief the pain.

Furthermore, high bioactivity score for nuclear receptor, GPCR and protease inhibitor is reflection of probable potential of this compound on these proteins and interestingly it needs further investigations.

Our computational results clearly demonstrate that the α-amyrins from *Cordia spp.* seed possess dual inhibitory effect on COX-2/5-LOX enzyme activity and would be effective as a complementary drug for human anti-inflammatory regime consumption. The identification and development of novel natural compounds with safe inhibitory activity is an urgent need of pharmaceutical industry for achieving more effective drugs especially for such incurable diseases like cancer.

## Figures and Tables

**Table 1:**
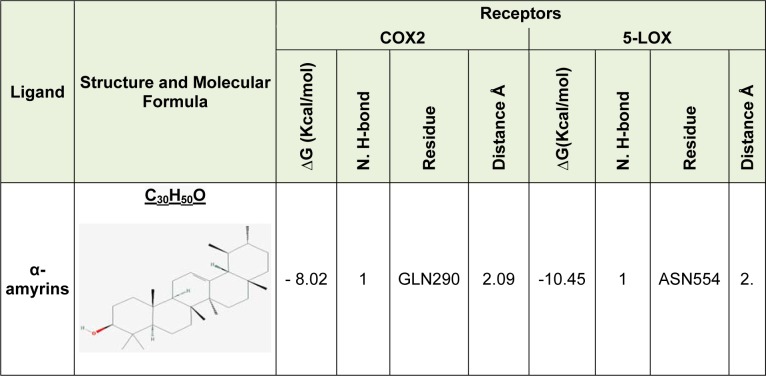
∆G (binding energy), hydrogen bonds along with their distances between the α-amyrins and active sites of COX2 and 5-LOX

**Table 2:**
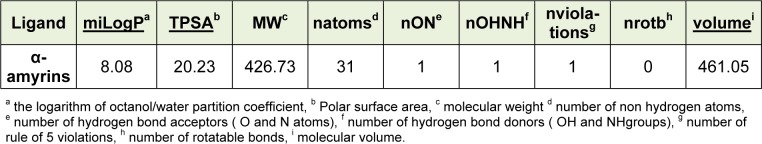
Molecular pharmacokinetic properties

**Table 3:**
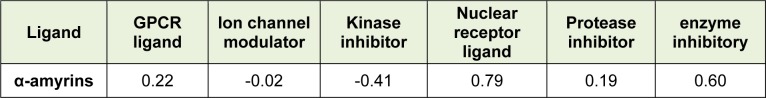
Bioactivity score (drug-likeness property) analysis of α-amyrins using Mol inspiration online software tool
